# Supplementary material for: Contingency and selection in mitochondrial genome dynamics
Source: eLife. 2022 Apr 11;11:e76557. doi: 10.7554/eLife.76557 (PMC9054137; doi:10.7554/eLife.76557)
Supplement: Transparent reporting form [file elife-76557-transrepform1.pdf]

1st Floor
24 Hills Road
Cambridge CB2 1JP, UK

P 01223 855340
W elifesciences.org
T @elife

eLife's transparent reporting form

We encourage authors to provide detailed information *within their submission* to facilitate the interpretation and replication of experiments. Authors can upload supporting documentation to indicate the use of appropriate reporting guidelines for health-related research (see EQUATOR Network), life science research (see the BioSharing Information Resource), or the ARRIVE guidelines for reporting work involving animal research. Where applicable, authors should refer to any relevant reporting standards documents in this form.

If you have any questions, please consult our Journal Policies and/or contact us: editorial@elifesciences.org.

Sample-size estimation

- You should state whether an appropriate sample size was computed when the study was being designed
- You should state the statistical method of sample size computation and any required assumptions
- If no explicit power analysis was used, you should describe how you decided what sample (replicate) size (number) to use

Please outline where this information can be found within the submission (e.g., sections or figure legends), or explain why this information doesn't apply to your submission:

Figure 1 and the accompanying results section describes the architecture of the experiment (replicates and sample size), and the motivation for this architecture which was to passage and sequence colonies until suppressivity (and mtDNA content) was homogeneous. Appropriate samples and sizes were determined empirically. An average sequencing coverage of ~700 per primary structure (described in methods) provides statistical power to identify breakpoints in mtDNA structure that are used in structure reconstruction.

Replicates

- You should report how often each experiment was performed
- You should include a definition of biological versus technical replication
- The data obtained should be provided and sufficient information should be provided to indicate the number of independent biological and/or technical replicates
- If you encountered any outliers, you should describe how these were handled
- Criteria for exclusion/inclusion of data should be clearly stated
- High-throughput sequence data should be uploaded before submission, with a private link for reviewers provided (these are available from both GEO and ArrayExpress)

Please outline where this information can be found within the submission (e.g., sections or figure legends), or explain why this information doesn't apply to your submission:

eLife Sciences Publications, Ltd is a limited liability non-profit non-stock corporation incorporated in the State of Delaware, USA, with company number 5030732, and is registered in the UK with company number FC030576 and branch number BR015634 at the address 1st Floor, 24 Hills Road, Cambridge CB2 1JP | August 2014

All data on technical/biological replicates is described either in figure descriptions or their accompanying results sections in the text.

No data at the level of sequencing samples was excluded, although spurious breakpoint signals in sequencing reads were excluded as described in methods.

Nanopore sequencing data from this study can be found at:

Reviewer URL:
https://datadryad.org/stash/share/SdnOZ3lghFUZVgl-RDmSb2kAA1Av5m45OwwCv4XJQNE.

Data DOI (Inactive until publication):
https://doi.org/10.5061/dryad.vdncjsxwx

Statistical reporting

- Statistical analysis methods should be described and justified
- Raw data should be presented in figures whenever informative to do so (typically when N per group is less than 10)
- For each experiment, you should identify the statistical tests used, exact values of N, definitions of center, methods of multiple test correction, and dispersion and precision measures (e.g., mean, median, SD, SEM, confidence intervals; and, for the major substantive results, a measure of effect size (e.g., Pearson's r, Cohen's d)
- Report exact p-values wherever possible alongside the summary statistics and 95% confidence intervals. These should be reported for all key questions and not only when the p-value is less than 0.05.

Please outline where this information can be found within the submission (e.g., sections or figure legends), or explain why this information doesn't apply to your submission:

(For large datasets, or papers with a very large number of statistical tests, you may upload a single table file with tests, Ns, etc., with reference to sections in the manuscript.)

Descriptions of statistical tests/metrics are found in figure descriptions or the corresponding results text. Bioinformatics analysis is described in methods, and suppressivity model selection statistics are found in the corresponding supplementary section as referenced in the text.

Group allocation

- Indicate how samples were allocated into experimental groups (in the case of clinical studies, please specify allocation to treatment method); if randomization was used, please also state if restricted randomization was applied
- Indicate if masking was used during group allocation, data collection and/or data analysis

Please outline where this information can be found within the submission (e.g., sections or figure legends), or explain why this information doesn't apply to your submission:

Colonies grouped into subfamilies in Figure 1 of the text are described in the corresponding results section.

eLife Sciences Publications, Ltd is a limited liability non-profit non-stock corporation incorporated in the State of Delaware, USA, with company number 5030732, and is registered in the UK with company number FC030576 and branch number BR015634 at the address 1st Floor, 24 Hills Road, Cambridge CB2 1JP | August 2014

Additional data files ("source data")

- We encourage you to upload relevant additional data files, such as numerical data that are represented as a graph in a figure, or as a summary table
- Where provided, these should be in the most useful format, and they can be uploaded as "Source data" files linked to a main figure or table
- Include model definition files including the full list of parameters used
- Include code used for data analysis (e.g., R, MatLab)
- Avoid stating that data files are "available upon request"

Please indicate the figures or tables for which source data files have been provided:

Nanopore sequencing data (that has been demultiplexed and labeled with the corresponding colony name in the main-text) is available alongside sequence alignment code and Python code for primary/alternate structure analysis below.<br><br>Reviewer URL:<br>https://datadryad.org/stash/share/SdnOZ3lghFUZVgl-RDmSb2kAA1Av5m45OwwCv4XJQNE.<br><br>Data DOI (Inactive until publication):<br>https://doi.org/10.5061/dryad.vdncjsxwx<br><br>Code DOI (Inactive until publication):<br>https://doi.org/10.5281/zenodo.5851771
